# HIV-1 escapes from N332-directed antibody neutralization in an elite neutralizer by envelope glycoprotein elongation and introduction of unusual disulfide bonds

**DOI:** 10.1186/s12977-016-0279-4

**Published:** 2016-07-07

**Authors:** Tom L. G. M. van den Kerkhof, Steven W. de Taeye, Brigitte D. Boeser-Nunnink, Dennis R. Burton, Neeltje A. Kootstra, Hanneke Schuitemaker, Rogier W. Sanders, Marit J. van Gils

**Affiliations:** Department of Medical Microbiology, Academic Medical Center, University of Amsterdam, 1105 AZ Amsterdam, The Netherlands; Department of Experimental Immunology, Academic Medical Center, University of Amsterdam, 1105 AZ Amsterdam, The Netherlands; Department of Immunology and Microbial Science and IAVI Neutralizing Antibody Center, The Scripps Research Institute, La Jolla, CA 92037 USA; Ragon Institute of MGH, MIT, and Harvard, Cambridge, MA 02139 USA; Janssen Pharmaceuticals, 2333 CN Leiden, The Netherlands; Department of Microbiology and Immunology, Weill Medical College, Cornell University, New York, NY 10065 USA

**Keywords:** HIV-1, N332, Envelope glycoprotein, Glycans, Broadly neutralizing antibodies, Variable regions, Cysteines

## Abstract

**Background:**

Current HIV-1 immunogens are unable to induce antibodies that can neutralize a broad range of HIV-1 (broadly neutralizing antibodies; bNAbs). However, such antibodies are elicited in 10–30 % of HIV-1 infected individuals, and the co-evolution of the virus and the humoral immune responses in these individuals has attracted attention, because they can provide clues for vaccine design.

**Results:**

Here we characterized the NAb responses and envelope glycoprotein evolution in an HIV-1 infected “elite neutralizer” of the Amsterdam Cohort Studies on HIV-1 infection and AIDS who developed an unusually potent bNAb response rapidly after infection. The NAb response was dependent on the N332-glycan and viral resistance against the N332-glycan dependent bNAb PGT135 developed over time but viral escape did not occur at or near this glycan. In contrast, the virus likely escaped by increasing V1 length, with up to 21 amino acids, accompanied by the introduction of 1–3 additional glycans, as well as 2–4 additional cysteine residues within V1.

**Conclusions:**

In the individual studied here, HIV-1 escaped from N332-glycan directed NAb responses without changing the epitope itself, but by elongating a variable loop that shields this epitope.

**Electronic supplementary material:**

The online version of this article (doi:10.1186/s12977-016-0279-4) contains supplementary material, which is available to authorized users.

## Background

The development of a safe and protective HIV-1 vaccine is a major challenge. Although progress has been made over 30 years of research, there is no immunogen that can efficiently elicit protective humoral immunity. The HIV-1 envelope glycoprotein spike (Env) on the viral membrane is the sole target for neutralizing antibodies (NAbs), and therefore designing an Env-based immunogen capable of inducing antibodies that can neutralize diverse globally circulating viral variants (broadly neutralizing antibodies; bNAbs) is an obvious vaccine strategy to pursue.

In 10–30 % of the HIV-1 infected individuals bNAbs develop, indicating that there are no insurmountable barriers for the induction of bNAbs by Env in humans [1–8]. Several passive immunization studies in non-human primates using bNAbs isolated from HIV-1 infected individuals have shown protection against HIV/SHIV acquisition, even with low bNAb doses and after repeated viral challenges [9–13]. Furthermore, 1 % of the HIV-1 infected individuals, termed “elite neutralizers”, develop exceptionally broad NAb responses, and some of these individuals develop a broad NAb response relatively quickly, i.e. within the first year after infection [8, 14]. Elite neutralizers might therefore serve as examples for Env-based vaccine design.

In infected humans, bNAbs appear to develop through co-evolution of HIV-1 Env and NAbs, probably via multiple pathways. In one scenario, iterative cycles of viral escape from (early) autologous NAbs and renewed NAb affinity maturation lead to NAb breadth [15–20]. This scenario is consistent with the large number of somatic mutations observed in HIV-1 bNAbs [21–24]. In a second scenario, escape from one NAb specificity can result in the exposure or creation of a bNAb epitope elsewhere on the Env surface, resulting in an independent bNAb lineage [25, 26]. Viral factors that have been associated with bNAb development include high viral load and antigenic diversity, prolonged antigenic stimulation and polyreactivity [1, 2, 27–30], but also specific Env characteristics on early viruses, such as short variable loops and lower glycan content [31–35].

Despite this knowledge, inducing bNAb responses by means of vaccination has proven a major challenge. In fact, even inducing consistent NAbs against the autologous, sequence-matched virus, with a vaccine based on a neutralization-resistant (Tier-2) primary isolate, has only been very recently achieved by immunizing with stabilized native-like trimers [36, 37]. The importance of viral evolution during Ab maturation, and in shaping bNAb responses, has led to the idea that sequential Env-based immunogens are required to steer Ab lineages towards becoming bNAbs. Indeed, immunogenicity studies have shown improved NAb responses when using sequentially isolated Envs from an SHIV_SF162p4_ infected macaque or from HIV-1 infected individuals who developed breadth, although NAbs were elicited only against neutralization-sensitive (Tier-1) viruses [38–40]. Therefore, studying Env evolution in infected individuals who eventually developed bNAbs, in particular elite neutralizers, can provide information that benefits sequential Env-based immunogen strategies.

The native, pre-fusion Env spike is a heterotrimeric complex of three gp120 subunits non-covalently linked to three gp41 subunits that are derived from a gp160 precursor protein through proteolytic cleavage [41]. Gp120 is composed of five conserved regions (C1–C5), interspersed with five exposed variable regions (V1–V5). C1–C5 form the gp120 core that is crucial for binding to target cells and transmitting receptor-induced conformational changes to the fusion machinery in gp41. V1–V5, in particular V1, V2 and V4, are highly diverse as a consequence of mutations, recombinations, deletions, and/or insertions. V1–V3 are important trimer association domains interacting at the apex of the trimer [42–46]. The high variability in these domains is driven by the need to continuously escape from NAbs and is facilitated by the high replication rate of the virus combined with the error prone reverse transcription process.

The *N*-linked glycans that are attached to 20–35 potential *N*-linked glycosylation sites (PNGS) on the backbone of gp120 account for 50 % of the mass of the external Env domains [47], and are usually not seen as foreign by the immune system. Therefore glycans and highly variable regions provide a formidable viral defense to protect the conserved Env regions from NAb attack [48–52]. Yet by necessity bNAb epitopes frequently incorporate glycan components, indicating that they are not completely immunosilent.

To fold and maintain its intricate structure gp120 typically has 18 cysteine residues that form 9 disulfide bonds, and one additional disulfide bond is present in gp41 [53, 54]. Disulfide bonds are key structural elements for protein folding and function, which also explains why disulfide bonds are usually conserved within protein families [54]. The positions of the disulfide bonds in gp120 are highly conserved across all isolates of HIV and SIV (Los Alamos database). Additional cysteine residues in the variable regions have been observed in HIV-1 (Table 1), but more so in HIV-2 and SIV (in particular in V2) [55–59].

**Table 1:** Relative occurrence of non-conserved cysteine residues in V1, V2 and V4 in HIV-1 subtypes

HIV-1 subtype	n^a^	V1 region				V2 region				V4 region			
		Canonical^b^	Plus 2 cysteines	Plus 4 cysteines	Total	Normal	2 cysteines	4 cysteines	Total	Canonical	Plus 2 cysteines	Plus 4 cysteines	Total
A	208	98.6	1.4	0	100	99.5	0.5	0	100	100	0	0	100
B	1338	94.4	5.5	0.1	100	99.9	0.1	0.1	100	99.9	0.1	0	100
C	952	94.5	5.1	0.3	100	99.7	0.3	0	100	99.5	0.5	0	100
D	101	99	1.0	0	100	100	0	0	100	100	0	0	100
F	39	100	0.0	0	100	100	0	0	100	100	0	0	100
G	61	73.8	26.2	0	100	100	0	0	100	100	0	0	100
Other	960	96.9	3.1	0	100	99.6	0.4	0	100	89.5	10.5	0	100
Total	3659	95.2	4.7	0.1	100	99.7	0.2	0	100	97.1	2.9	0	100

^a^The number of Env sequences used to calculate the percentage of viruses with canonical or non-conserved cysteine residues per subtype

^b^The percentage of viruses with normal or non-conserved cysteine residue

Here we have studied Env evolution in response to NAb pressure over time in an individual from the Amsterdam Cohort Studies on HIV-1 infection and AIDS (ACS) infected by intravenous drug use (IDU), who developed an elite bNAb response within 30 months post-serconversion (post-SC) [14]. NAb responses developed against autologous and heterologous viruses and were strongly dependent on the glycan at position 332. Viral escape from these responses did not occur at or near the N332-glycan, but rather involved the elongation of V1, by up to 21 additional amino acids. This V1 extension coincided with the introduction of 2–4 additional cysteine residues and 1–3 glycans, and was accompanied by a fitness loss. These findings provide insights into how HIV-1 can escape from N332-directed bNAb responses without changing the epitope itself.

## Results

### Development of autologous NAb responses in elite neutralizer D16916

**Fig. 1:**
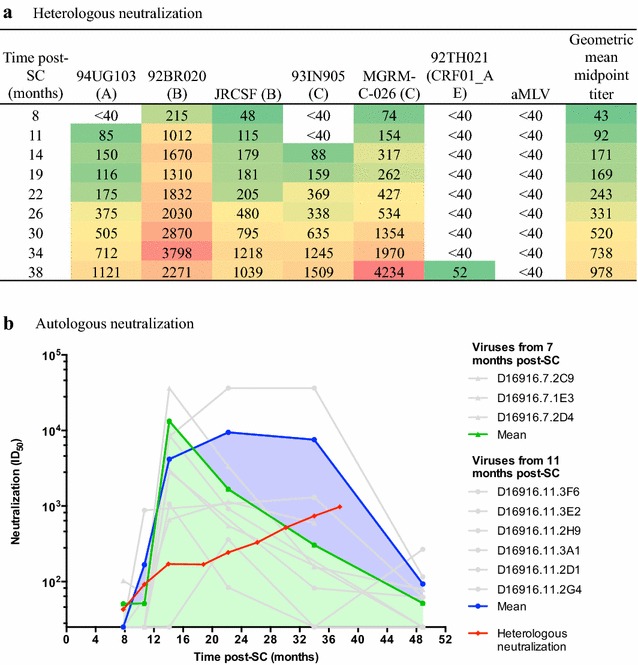
Development of heterologous and autologous NAb responses in elite neutralizer D16916. **a** Neutralizing activity over the course of HIV-1 infection against the individual viruses of a 6-virus panel that is representative for HIV-1 variation worldwide [8, 32]. The ID_50_ against each virus as well as the geometric mean midpoint titer for all 6 viruses combined are given for D16916 sera taken 8, 11, 14, 19, 22, 26, 30, 34 and 38 months post-SC. A color scale is used to indicate low (*green*) to high (*red*) ID_50_ values. **b** Longitudinal neutralization of autologous viruses. The mean ID_50_ values of longitudinal sera (x-axis) against the autologous viruses (3 for month 7 and 6 for month 11, all in *grey*) are plotted in *green* (month 7) and *blue* (month 11). The *red line* indicates the geometric mean midpoint titer against the heterologous 6-virus panel from **a** [14]

Individual D16916 entered the Amsterdam Cohort Studies (ACS) HIV-1 negative and seroconverted during active follow-up after infection with a clade B virus. In the first five years post-SC, this individual had stable CD4^+^ T cell numbers (average 395 cells/μl) and low to undetectable viral loads (Additional file 1: Fig. S1). We previously described the development of the bNAb responses in this individual [14]. At 38 months, D16916 qualified as an elite neutralizer, neutralizing all viruses of a six-virus panel that is representative of global HIV-1 variation, at a geometric mean midpoint titer of 978 (Fig. 1a). By this standard this individual has the broadest and most potent neutralization observed in the ACS. Furthermore, neutralization of multiple heterologous Tier-2 viruses was observed at around 8 months, and broad neutralization, defined as a geometric mean midpoint titer of >100, was observed around 11 months, which is unusually early [14].

To gain more insight into the unusually rapid heterologous NAb response, we first studied the development of the autologous NAb response at and preceding the 11 month time point (Fig. 1b). Therefore, we tested three and six viral isolates from 7 and 11 months post-SC, respectively. We observed low neutralizing activity in serum from 8 months against one out of three viruses from 7 months, and no activity against any of the other viruses from 7 and 11 months tested. Serum neutralization against the month 7 viruses rapidly increased until it peaked at 14 months, after which it declined very rapidly. The increase in neutralizing activity could also be observed against viruses from 11 months, although the activity against these viruses remained rather stable between 14 and 34 months before declining. Thus, around a year post-SC, i.e. when heterologous NAb responses first appeared, this individual had developed strong autologous NAb responses against the viruses that were present at 7 months post-SC. Whether earlier autologous NAb responses against the preceding viruses were present, could not be tested because such samples were not available for analysis.

### N332-directed bNAb responses in elite neutralizer D16916

Five viruses of the six-virus panel were neutralized by serum from individual D16916 as early as 14 months after infection. The 92TH021 virus was an exception and was only very weakly neutralized by month 38 (Fig. 1a). We noted that the five sensitive viruses had the PNGS at position 332, which is frequently targeted by bNAb responses [60], whereas 92TH021 contains the PNGS at position 334. To assess whether the bNAb response in individual D16916 was N332-glycan dependent, we measured the neutralization activity of serum from 14 months against three viruses of the six-virus panel in comparison with their N332A glycan knock-out variants: JRCSF, 92BR020 (both clade B viruses) and MGRM-C-026 (a clade C virus) [61]. Unfortunately, changing the glycan from position 334–332 in 92TH021 did not yield an infectious virus. We observed a marked decrease in neutralization sensitivity for the three N332A variants, when compared with the wild-type (WT) viruses (Table 2). We also tested serum from 30 months, when elite bNAb activity was present, against the Tier-2 subtype A BG505 virus, which naturally lacks the N332-glycan, as well as the BG505 T332N glycan knock-in mutant [62]. While the WT BG505 virus was not neutralized (10 % neutralization at a 1:40 dilution), we did observe neutralization of the T332N glycan knock-in virus (77 % neutralization at a 1:40 dilution; see also Table 2). These data suggest that a substantial proportion of the bNAb response in elite neutralizer D16916 is directed against the N332-glycan.

**Table 2:** N332-directed heterologous NAb responses in elite neutralizer D16916

	ID_50_^a^		Fold^d^ reduction
	WT^b^	N332A	
92BR020	1711	49	35
JRCSF	211	60	3.5
MGRM-C-026	269	70	3.9

	T332N^c^	WT (T332)	
BG505	63	<40	>1.5

^a^50 % inhibitory dilution (ID_50_)

^b^ID_50_ of month 14 serum tested against the 92BR020 and JRCSF (both clade B) and MGRM-C-026 (clade C) viruses and their N332A mutants

^c^ID_50_ of month 30 serum tested against the BG505 virus (clade A) and its T332N mutant

^d^The fold-reduction in neutralization as a consequence of the lack of the N332-glycan

### Development of viral resistance to N332-directed bNAb PGT135, as well as bNAbs b12 and 12A21

**Fig. 2:**
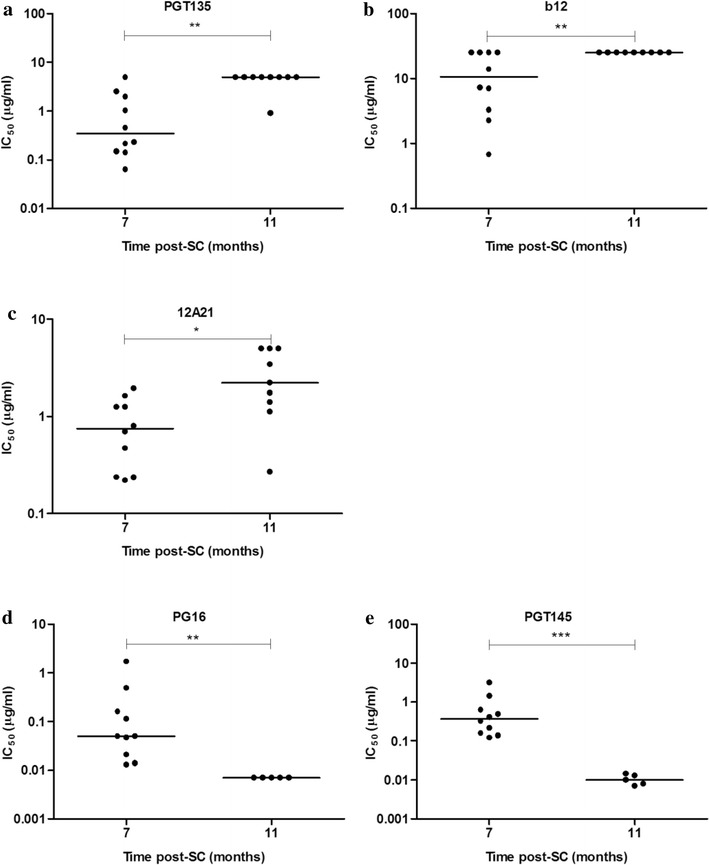
Sensitivity of viral isolates from individual D16916 to bNAbs. Clonal HIV-1 variants from 7 and 11 months post-SC were tested for their neutralization sensitivity against bNAbs PGT135 (**a**), b12 (**b**), 12A21 (**c**), PG16 (**d**) and PGT145 (**e**) and grouped according to their target epitope OD-glycan, CD4-bs and V1V2 apex. The *graphs* show IC_50_ values for each virus isolate, as determined by linear regression. Differences were considered statistically significant when p values were ≤ 0.05, represented by *asterisks* (*p ≤ 0.05; **p ≤ 0.005, ***p ≤ 0.001). The *horizontal bars* represent the median IC_50_ value per time point

To study the effect of the N332-directed NAb response on the viral evolution in individual D16916 we tested longitudinally isolated viruses for their sensitivity to N332- directed bNAbs PGT121, PGT126, PGT128, PGT135 and 2G12. No differences were observed in the neutralization sensitivity of viruses from month 7 and 11 to the N332-directed bNAbs, except for PGT135 (Fig. 2a; Additional file 2: Fig. S2A–D). While, 9 out of 10 viruses isolated from month 7 were sensitive to PGT135, only 1 out of 9 viruses from month 11 was sensitive to PGT135. Thus, from month 7 to month 11, the majority of viruses acquired resistance to the N332-directed bNAb PGT135 (>14-fold when comparing the median 50 % inhibitory concentration (IC_50_) values, p = 0.0016). In addition, we also tested bNAbs targeting other epitopes. We observed, to a lesser extent, development of resistance against the CD4bs-directed bNAbs b12 and 12A21 (2.5-fold, p = 0.0072 and 3-fold, p = 0.01, respectively; Fig. 2b, c). In contrast, we observed an increased sensitivity to bNAbs PG16 and PGT145, directed against a quaternary glycan-dependent V2 epitope (by >7-fold, p = 0.0027 and >36-fold, p = 0.0007, respectively; Fig. 2d, e). We did not find significant differences in the neutralization sensitivity to bNAbs VRC01, PG9, and 8ANC195 (Additional file 2: Fig. S2E–G).

### Lack of viral escape mutations in the N332-directed bNAb epitope

**Fig. 3:**
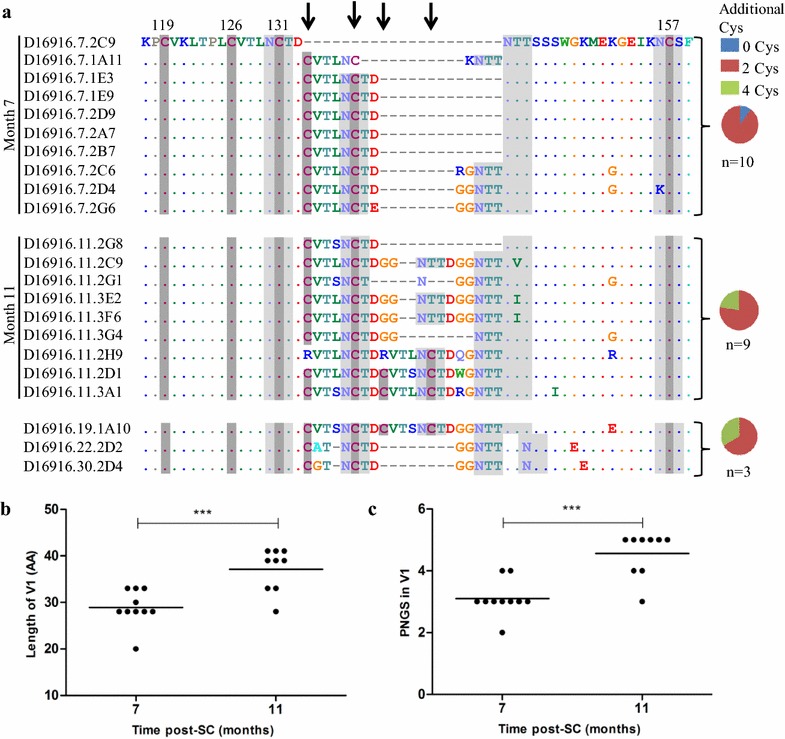
Extension of V1 and insertion of unusual disulfide bonds. **a** Amino acid alignment of the D16916 V1 loop of clonal HIV-1 variants isolated over time. Cysteine residues are indicated in *dark grey boxes* and potential N-linked glycans in *light grey*. The frequencies of additional cysteines (0: *blue*, 2: *red*, 4: *green*) in the sequences from viruses isolated at 7, 11 and 19, 22 and 30 months post-SC combined are shown in the pie charts. HXB2 numbering has been used to annotate the positions of the conserved cysteine residues. *Arrows* indicate the positions of the introduced cysteine residues. **b** The length of V1 and **c** the number of PNGS in V1 of clonal HIV-1 variants over the course of infection are shown. In both *panels*, the *horizontal bars* represent the mean values per time point tested and differences were considered statistically significant when p values were ≤ 0.05, represented by *asterisks* (***p ≤ 0.001)

Since we observed the development of viral resistance to PGT135, b12 and 12A21, we studied the evolution of the epitopes of these bNAbs. We sequenced the complete gp160 *env* from multiple viruses from different time points (Fig. 3a). Phylogenetic analyses showed that, with one exception, the month 7 and month 11 sequences formed separate clusters (Additional file 3: Fig. S3). Interestingly, we did not observe escape mutations at the N332-glycan, nor did we find escape mutations anywhere else in the PGT135 epitope (Additional file 4: Fig. S4A), suggesting that changes outside the PGT135 epitope were responsible for the increase in PGT135-resistance from month 7 to 11.

We did observe mutations in the b12 and 12A21 epitopes at month 11 compared to month 7, notably at position 475, a contact residue for b12, and at position 462, a contact residue for 12A21 (Additional file 4: Fig. S4B). However, none of these changes were associated with increased resistance to b12 or 12A21, when sensitivity of specific viral clones and their sequences were compared. The increase in neutralization sensitivity for PG16 that developed from month 7 to 11 could also not be explained by mutations in the known epitope, as there were none (Additional file 4: Fig. S4C).

### Elongation of V1 with insertions of unusual disulfide bonds

Since we did not find mutations in the bNAb epitopes that could explain the increase in neutralization resistance we studied the evolution of the entire gp160 sequence. We identified many amino acids that changed from month 7 to month 11 and these amino acids were scattered across the gp160 sequence. Some of these changes became fixed in the viral population, but none of them stood out as likely candidates to explain the altered neutralization profile.

However, we observed a significant increase in the length of gp160 and the number of PNGS from month 7 to month 11 (mean of 827 vs. 836 amino acids, p = 0.0009, mean of 30 vs. 32 PNGS, p < 0.0001, respectively). Both were mostly attributable to an increase in V1 length (mean of 29 vs. 37 amino acids, p = 0.0006, mean of 3 vs. 4.5 PNGS, p = 0.0001, respectively; Fig. 3b, c). The additional PNGS in the V1 were all NXT motifs, which have a higher probability to become glycosylated compared to NXS motifs [63–65].

At 7 months, we found one virus with a short V1 (20 amino acids) while the other 9 sequences showed V1 elongation by 8, 10, or 13 additional amino acids, including one or two extra PNGS (Fig. 3a). The elongation of the V1 was likely caused by an 8 amino acid duplication of the sequence CVTLNCTD containing 2 extra cysteines and one PNGS (5 isolates), followed by the insertion of an additional 5 amino acids (GGNTT or RGNTT), including an additional PNGS (3 isolates). The additional 2 cysteines are likely to pair and form a disulfide bond in an “oven mitt” structure (Additional file 5: Fig. S5B) [59].

At 11 months we observed three isolates with similar features as described for the isolates from 7 months containing 2 additional cysteines. Remarkably, in three other isolates the V1 length was further increased (to 39 amino acids), with a 6 amino acid duplication of the sequence DGGNTT. Interestingly, in three other virus isolates the 8 amino acid sequence CVTNLCTD was again duplicated and further mutated, resulting in a V1 with 4 extra cysteines (2 isolates), while in the last virus isolate the first cysteine of each 8 amino acid repeat was replaced by an arginine, restoring the number of cysteines to +2 (Additional file 5: Fig. S5C). The 4 extra cysteines might form 2 extra cysteine bridges, with “oven mitt” structures, but an alternative structure is now also possible (Additional file 5: Fig. S5D–F). The longest V1 sequences (clones 2H9, 2D1 and 3A1 at month 11 and clone 1A10 at month 19) have a V1 of 41 amino acids, i.e. 21 amino acids longer than the shortest V1 (clone 2C9 at month 7). For comparison, the average V1 length in Env sequences in the Los Alamos Database is 27 amino acids, with 95 % of the sequences falling in the range of 15–39 amino acids, illustrating that a V1 of 41 amino acids is unusually long.

We could only isolate one virus from 19, 22 and 30 months (Fig. 3a). The virus isolated at 19 months had a V1 sequence that was very similar to the unusually long month 11 V1 containing 4 extra cysteines, while the month 22 and 30 sequences contained moderately long V1 sequences with two extra cysteine residues (Fig. 3a).

We analyzed the frequency of additional cysteines in V1, as well as V2 and V4 in natural HIV and SIV isolates. The presence of additional cysteines in V1 is common in HIV-2 and SIV (Los Alamos Database), but rare in HIV-1 (Table 1). 4.7 % of HIV-1 isolates contain two extra cysteines in V1, while 4 extra cysteines are present in only 0.1 % of HIV-1 sequences, illustrating that the Env protein in individual D16916, who developed elite NAb responses, evolved to have rather unusual properties.

### Involvement of V1 length in resistance to bNAbs PGT135, b12 and 12A21

**Fig. 4:**
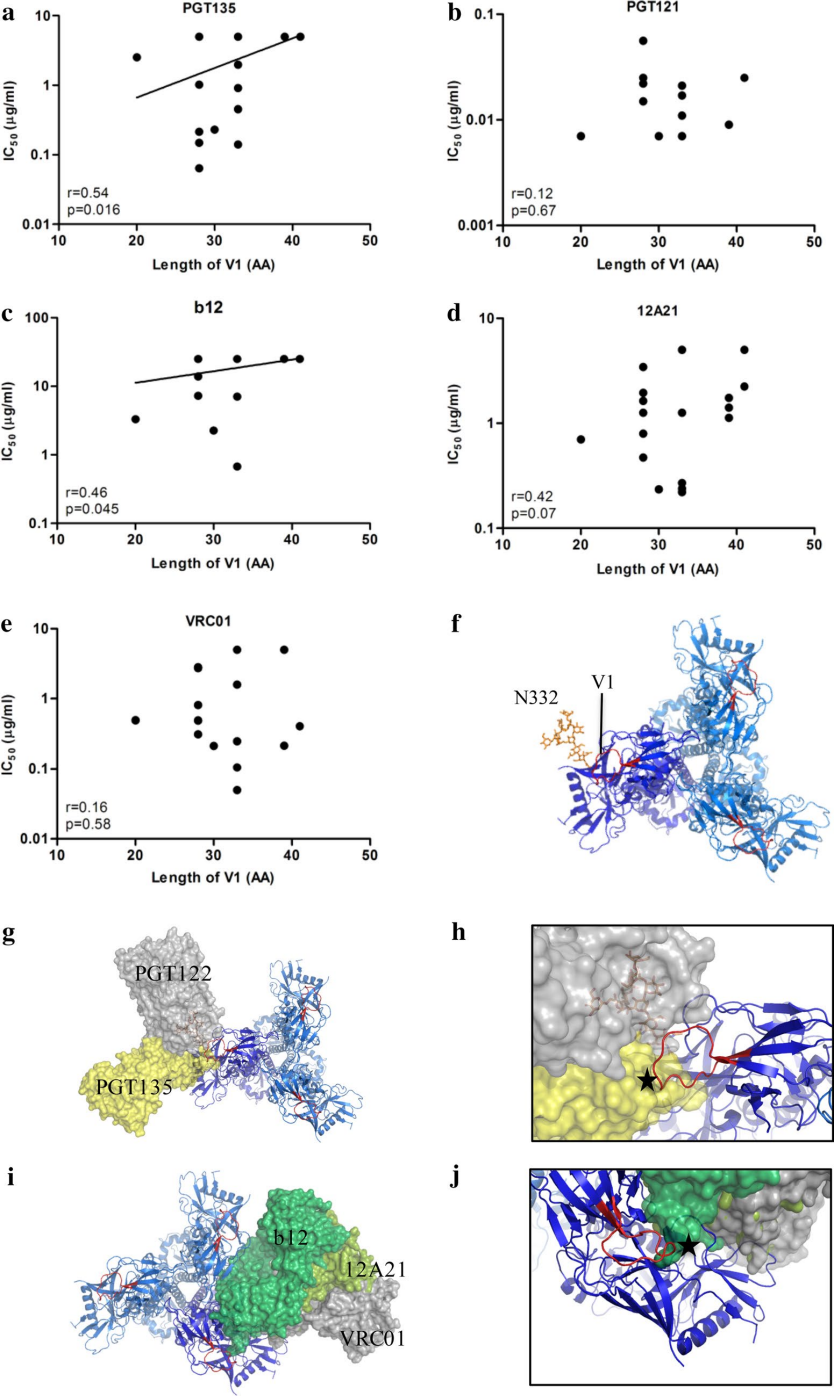
Involvement of V1 length in resistance to bNAbs PGT135, b12 and 12A21. Correlation plots between the V1 length and neutralization sensitivity for mAbs PGT135 (**a**), PGT121 (**b**), b12 (**c**), 12A21 (**d**), and VRC01 (**e**). The r and p values for the linear regression are given. For correlations that were statistically significant, the regression line is shown. (**f**) Top view of the Env trimer with one protomer shown in *dark blue* and the other protomers in *light blue*. The V1 loops are indicated in *red* and the N332-glycan is shown on one protomer. **g** View of the trimer in the same orientations as **f** in complex with PGT135 (*yellow*) and PGT122 (*gray*). We used PGT122 in our figure instead of PGT121 since the HIV-1 Env trimer structures were solved in complex with PGT122, whereas for PGT121 the structure was only solved in complex with a glycan [42] or recently a complex of a PGT121 precursor in complex with the HIV-1 Env trimer [120]. It was shown that PGT121 and PGT122 bind to the Env protein with the same angle of approach in a very similar way. **h** Detailed view of the expected clash of PGT135 with the V1 loop indicated by an *asterisk*. **i** View of the trimer in the same orientations as **f** in complex with b12 (*green*) and 12A21 (*dark green*) and VRC01 (*gray*). **j** Detailed view of the expected clash of b12 with the V1 loop indicated by an *asterisk*. The figures were drawn using pymol (www.pymol.org) by aligning the gp120 structures of 4JM2.pdb (gp120 plus PGT135; PMC3823233 [60]), 2NY7.pdb (gp120 plus b12: PMC2584968 [121]), 4JPW.pdb (gp120 plus 12A21; PMC3792590 [122]), and 3NGB.pdb (gp120 plus VRC01; PMC2981354 [123]) with the *dark blue* protomer of the BG505 SOSIP.664 trimer in complex with PGT122 and 35O22 (4TVP.pdb [43])

The elongation of the V1 from month 7 to 11, and the accompanying introduction of additional cysteine residues, led us to speculate that the V1 was responsible for the increased resistance against bNAbs PGT135, b12 and 12A21. For PGT135, we observed a statistically significant positive correlation between V1 length and neutralization resistance (r = 0.54, p = 0.016; Fig. 4a). Inspection of the Env structure in complex with PGT135 reveals that a longer V1 region could indeed clash with PGT135 (Fig. 4g, h). For PGT121, which also targets the N332-glycan, we did not observe a correlation between the length of the V1 region and neutralization sensitivity (Fig. 4b), which might be related to the different angle of approach of the bNAb, thereby avoiding a clash with V1 (Fig. 4h). In addition, we observed a significant positive correlation between V1 length and neutralization resistance for b12 and a trend for 12A21, both directed against the CD4bs (r = 0.46, p = 0.045 and r = 0.42, p = 0.07, respectively; Fig. 4c, d). These bNAbs can also clash with unusually long V1 regions (Fig. 4i, j), while VRC01, another CD4bs bNAb for which no correlation between the length of V1 and neutralization sensitivity was found (Fig. 4e), targets the CD4bs with an angle that avoids the V1 (Fig. 4i, j).

### Association of V1 length with loss of viral fitness

**Fig. 5:**
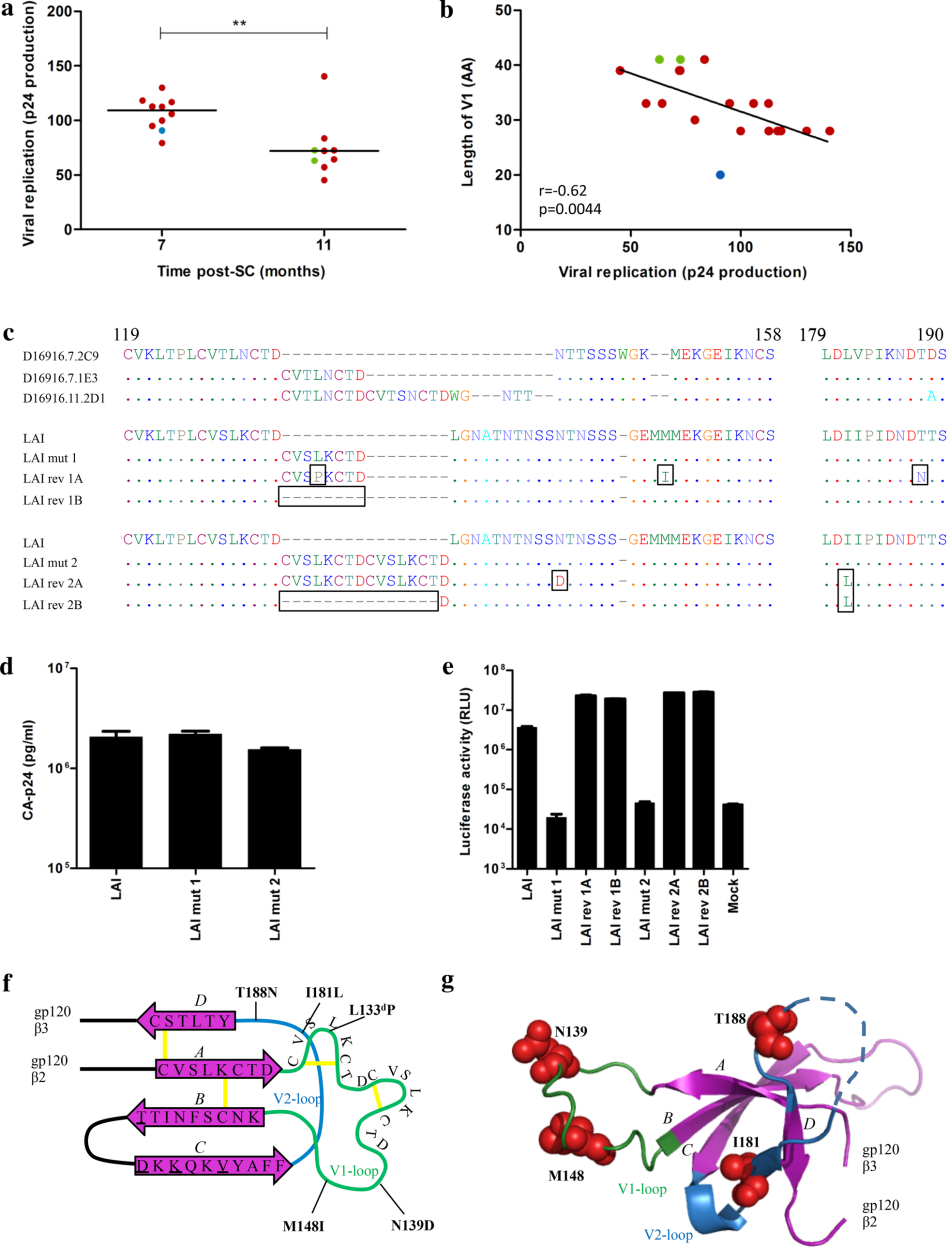
Accommodation of long V1, associated with viral fitness loss, by compensatory mutations. **a** Viral replication of clonal viral isolates from month 7 versus month 11 with the horizontal bars representing the median p24 values over the viruses isolated at that time point. **b** Linear regression between viral replication, expressed as p24 production per day, and V1 length for viruses from month 7 and 11. Virus isolates are *colored* based on number of additional cysteine residues in their V1 (0: *blue*, 2: *red*, 4: *green*). **c** V1 sequence alignment of representative virus isolates from individual D16916 with 0, 2 and 4 additional cysteines (clones D16916.7.2C9, D16916.7.1E3, D16916.11.2D1), as well as HIV-1_LAI_, HIV-1_LAI_ mutants 1 and 2, and their revertants. **d** CA-p24 ELISA of HIV-1_LAI_ and mutant virus stocks, produced by transient transfection of HEK293T cells. **e** TZM-bl cells were infected with 500 pg CA-p24, and infectivity was measured after 48 h infection. **f** Schematic V1/V2 topology. β-strands are depicted as *purple arrows* and disulphide bonds as *yellow lines*. The V1 and V2 loop are indicated in *green* and *blue lines*, respectively. The substitutions designed to restore the epitopes for bNAbs PG9 and PG16 are underlined and the locations of the HIV-1_LAI_ reversions are indicated in *bold*. **g** Ribbon diagram of **f** with the position of the HIV-1_LAI_ reversions indicated as *red spheres* (except L → P at the fourth position of the insert) and labeled according to their position in the linear sequence. Note that the elongated V1 with additional cysteine residues is not depicted

Based on the low occurrence of long V1 sequences with extra disulfide bonds in natural isolates, we hypothesized that the elongation of the V1 and the inclusion of extra cysteine residues, possibly a consequence of NAb pressure, might have a negative impact on viral fitness. We analyzed the replication kinetics of all virus isolates from 7 and 11 months on phytohemagglutinin (PHA) stimulated peripheral blood mononuclear cells (PBMCs). We observed that there was a significant difference between the replication rates of the viral isolates from 7 and 11 months (p = 0.0041, Fig. 5a), and a significant negative correlation between the replication kinetics and the V1 length (r = −0.62, p = 0.0044; Fig. 5b). Thus, viruses isolated later during infection harboring longer V1 had lower replication rates compared to earlier viruses harboring a shorter V1. These data support the hypothesis that the virus present in individual D16916 was under selection pressure from NAbs to generate and preserve gp160 proteins with unusually long V1 loops containing additional cysteines that were accompanied with a decrease in gp160 function.

### Accommodation of elongated V1 loops with additional cysteine bonds in a different virus isolate

To answer the question whether the long V1 sequences with additional cysteine residues were isolate-specific or could be incorporated into an unrelated HIV-1 strain, we investigated the infectivity of variants of the HIV-1_LAI_ isolate (containing 4 substitutions to facilitate PG9/PG16 binding: S162T, G167D, V169K and E172V) in which the V1 sequences were extended with 8 or 16 amino acids, based on the increase in V1 length observed in individual D16916. In mutant 1, the V1 was extended by 8 amino acids (CVSLKCTD; based on month 7 isolate D16916.7.1E3) and in mutant 2 by 16 amino acids (CVSLKCTDCVSLKCTD; based on month 11 isolate D16916.11.2D1), harboring 2 or 4 extra cysteines, respectively (Fig. 5c). Virus stocks were generated in HEK293T cells and the two mutant viruses were analyzed for their ability to infect TZM-bl cells. We observed that mutant 1 and 2 produced similar amounts of capsid (CA)-p24 antigen compared to wild-type virus (Fig. 5d), but were not able to infect TZM-bl cells (Fig. 5e). These data suggest that the long V1 sequences cannot be introduced in any virus isolate and/or that compensatory changes are required to do so.

Next we performed in vitro virus evolution experiments using the HIV-1_LAI_ mutant. Four independent cultures of SupT1 cells were transfected with the molecular clones of either HIV-1_LAI_ mutant 1 (cultures 1A and 1B) or mutant 2 (cultures 2A and 2B). The transfected cells were maintained for 3 months, and the cultures were inspected every week by eye for the presence of syncytia and by CA-p24 enzyme linked immunosorbent assay (ELISA). For mutant 1, replicating virus was identified 2 and 3 weeks post-transfection (cultures 1B and 1A, respectively), whereas for mutant 2 replication was observed after 5 weeks (both cultures). In contrast to the original mutant viruses, the evolved viruses obtained after 3 months of culturing were able to infect TZM-bl cells (Fig. 5e). Both HIV-1_LAI_ and the evolved viruses were resistant to all N332-dependent bNAbs as well as the apex bNAbs, precluding analyses to study the differences in sensitivity to N332-directed specificities in the context of the HIV-1_LAI_ isolate (data not shown).

Total cellular DNA was extracted from the infected cells at 3 months post-transfection. The *env* sequences revealed mutations in all four cultures (indicated by boxes; Fig. 5c). In culture 1A the 8 amino acid insertion was maintained, but a substitution was observed at the fourth residue of the inserted sequence (L133^d^P), as were substitutions further downstream in V1 and V2 (M148I and T188N). In the 1B culture we observed the deletion of the introduced 8 amino acids. In culture 2A we found two substitutions: N139D in V1 and I181L in V2, whereas in culture 2B we observed the deletion of all 16 introduced amino acids except for one aspartic acid, as well as an I181L substitution.

Interestingly, we found that the I181L substitution was present in all the viral isolates from individual D16916, suggesting that the presence of a leucine at position 181 might facilitate the V1 insertion. Furthermore, the T188N substitution that emerged during evolution in culture 1B was also observed in one viral isolate (data not shown). The 181 and 188 residues are located at the base of V2 that interacts with the base of V1 and they might therefore contribute to the appropriate positioning of the elongated V1 (Fig. 5f, g).

## Discussion

To guide Env-based vaccine design, it is important to understand the mechanisms that are involved in the development of bNAb responses and viral escape from them. Here we studied virus evolution in individual D16916 of the ACS with unusually potent and broad NAb responses, which developed much faster than usually seen, i.e. within 1 year as opposed to 2–3 years [14], making individual D16916 a particularly attractive case to inform vaccine design.

In concordance with other studies, we observed that the autologous NAb responses in individual D16916 developed in parallel with the heterologous NAb response development [2, 17, 18, 20, 26, 51, 66, 67], supporting the view that iterative cycles of NAb escape and renewed affinity maturation lead to increased neutralization breadth [16–20]. When dissecting the heterologous NAb response we observed that this response was dependent on the glycan at position 332 of Env. This glycan is a central feature of the supersite of immune vulnerability on the glycosylated outer domain of gp120 that is targeted by multiple bNAbs [60], and is commonly targeted by NAb responses in HIV-1 infected individuals [68]. Moore et al. showed that in two individuals the appearance of the N332-glycan on Env, which allowed immune escape early during infection, created a new epitope on the autologous virus that triggered the development of N332-directed bNAbs [25]. We were not able to generate viruses from before 7 months post-SC, and therefore we cannot determine whether a similar pathway was followed in individual D16916.

Various known N332-directed bNAbs have been isolated from different HIV-1 infected individuals and are derived from different germline lineages. Extensive studies showed that most of these bNAbs interact differently with the N332-glycan, even when the bNAbs are of the same antibody lineage (such as PGT121, PGT122 and PGT124), probably due to different maturation pathways [69, 70]. In addition to the targeting of the N332-glycan, these bNAbs often also recognize other glycans, such as N137, N156, N301, as well as protein backbone. In individual D16916 we observed viral escape from N332-directed bNAb PGT135 [60] but not from other N332-glycan targeting bNAbs, suggesting that PGT135-like N332-directed bNAbs were elicited by this individual.

Escape from N332-directed (b) NAbs has been reported, both in HIV-1 infected individuals and in infected macaques and humanized mice that received N332-directed bNAbs as immunotherapy. Such escape is usually mediated via a direct mutation of the N332-glycan or mutations elsewhere in the target epitope [25, 71–75]. In contrast, in individual D16916 it appears that viral escape from N332-directed bNAbs was mediated by a large increase in V1 length, rather than direct mutation of the N332-glycan or residues nearby. Escape via elongation of V1, in combination with mutations in other variable regions, but not the removal of the N332-glycan, was also observed in one rhesus macaque that was inoculated with SHIV_AD8-LN_ and developed potent bNAbs targeting the N332-glycan [76, 77]. However, the elongated V1 (by 6 amino acids) was not the principal determinant of viral resistance in this animal, as it was shown that mutations in V3 were responsible for the escape [72]. The viral escape in individual D16916 from N332-directed, PGT135-like NAb responses through V1 masking is supported by the positive correlation between the V1 length and the increased resistance to PGT135. Multiple studies have described that an increase in variable loop length is a shielding mechanism to protect other bNAb epitopes from antibody recognition [33, 51, 78–88]. The viral escape from N332-directed bNAbs, and to a lesser extent resistance to CD4bs bNAbs, via an elongated and stabilized V1, could be achieved by intraprotomer or interprotomer masking of the N332 and/or CD4bs, and both scenarios are compatible with the trimer structure (Fig. 4) [43, 89–92].

Remarkably, we observed that the elongation of the V1 was accompanied by the introduction of additional cysteine residues, which is a rare phenomenon. As some studies suggest that viral escape from autologous NAbs can result in reduced viral replication, whereas others observed minimal fitness cost [31, 67, 93–97], we were interested if an elongated V1 with additional cysteine residues could indeed reduce viral replication. We did not find a significant difference in replication rate or neutralization sensitivity for bNAbs when comparing viruses that harbored 0, 2 or 4 extra cysteines in the V1 (data not shown), however longer V1 loops did correlate with reduced replication rates. Rather than being a mechanism of immune escape by itself [59], the additional cysteine bonds might therefore be coincidental, through the duplication of an already existing part of V1. The extra cysteines might also have facilitated the presence of the long V1 loops by stabilizing its extended structure, because if they were disadvantageous, they would probably have disappeared from the viral population. Although an extended V1 harboring additional cysteines is not observed frequently, incorporating such V1 loops into HIV-1_LAI_ showed that HIV-1 can cope with these features via the introduction of compensatory amino acid substitutions in and around the inserted sequence (cultures 1A: L133^d^P, M148I and T188N, and 2A: N139D and I181L; Fig. 5). Thus, while long V1 loops with additional disulfide bonds have adverse effects on Env function, sometimes leading to their deletion, they can also be accommodated by the presence of compensatory changes within the inserted segment or in close proximity of the insertion in V1 or V2. The mutations observed in culture 1A and 2A were also detected in viruses isolated from D16916 and therefore seem to facilitate the elongated V1.

## Conclusions

In summary, despite the limited availability of viral samples for our study subject, our results illustrate that HIV-1 can escape from N332-directed NAb responses in a time frame of 4 months, not by changing the epitope, but by elongation of the V1 loop. The results are noteworthy in light of sequential, patient-based vaccine strategies to steer desirable Ab lineages that have the potential to become bNAbs [17, 18, 98]. Immunizing with sequential immunogens from individual D16916 will most likely not lead to development of N332-directed bNAbs when Env’s with the long V1 loops are included, because in those immunogens the desired target epitope is masked. On the other hand, these same Env’s more efficiently present the bNAb epitopes located at the trimer-apex. We conclude that longitudinal Env sequences should be studied in detail before using them in sequential immunization strategies.

## Methods

### ACS participant D16916

Individual D16916 is a male participant of the ACS who was infected in 1990 with HIV-1 subtype B, likely via IDU. He entered the ACS as HIV-1 negative and seroconverted during active follow-up, and was initially described in studies that investigated the number of ACS individuals with broad neutralization (van den Kerkhof et al. manuscript in preparation), and the longitudinal development of bNAbs in elite neutralizers ([14]; individual IDU1 in that study). D16916 was under observation for more than 12 years and until 5 years post-SC this individual had constant CD4^+^ T-cell numbers (average 395 cells/μl), had low/undetectable viral loads, did not receive anti-retroviral therapy and had no clinical signs of AIDS (Additional file 1: Fig. S1). In addition, this individual had no known protective human leukocyte antigen (HLA) type but was heterozygous for the Δ32 deletion in the CCR5 gene. Serum and PBMC samples were taken approximately every 4 months. Here we studied clonal virus isolates from 7.4, 10.7, 18.8, 22.2 and 30.1 months post-SC. For autologous neutralizing responses, we used serum samples from 7.8, 10.7, 14.1, 22.2, 34.0 and 48.9 months post-SC, whereas for heterologous neutralizing responses we used serum samples from 7.8, 10.7, 14.1, 18.8, 22.2, 26.2, 30.1, 34.0 and 37.5 months post-SC. An overview can be found in Additional file 1: Fig. S1. Rounded numbers were used in the text to refer to these time points. Due to the low viral load and limited serum availability, we were unable to extract multiple clonal virus isolates from the last three time points, as described below, or to test both autologous and heterologous neutralizing responses with sera from the same time points for all viruses.

### Virus isolation from PBMCs

PBMC samples from individual D16916 were collected at month 7, 11, 19, 22 and 30 post-SC. Single clonal virus variants were isolated from selected PBMCs by direct limiting dilution of the cells. Cells were co-cultivated with PHA-stimulated PBMCs from ten healthy HIV-1 uninfected donors, as described previously [99, 100]. To prevent sequence changes during in vitro culture, the number of passages in PBMCs was kept to a minimum. An earlier study showed that the quasispecies of clonal viral variants isolated from PBMCs are highly similar to sequences from viral RNA in plasma samples from the same individual [101]. We were able to generate ten and nine replication competent clonal virus variants at 7 and 11 months post-SC, respectively, and only one viral variant at 19, 22 and 30 months post-SC, probably due to low viral loads. Furthermore, phylogenetic analyses suggested that those viruses might be archived viruses (Additional file 1: Fig. S1, Additional file 2: Fig. S2).

### Gp160 sequence analysis

Proviral *env* genes from PBMCs that were infected in vitro with a single clonal HIV-1 variant were PCR-amplified and sequenced [102–104]. Nucleotide sequences were aligned using ClustalW in the software package of BioEdit [105], and edited manually, excluding contamination. The gp160 sequences were used to construct a Maximum Likelihood (ML) tree. The best-fit nucleotide substitution model (GTR + I+G), selected by hierarchical likelihood ratio tests (hLRTs, Model Test 3.7 [106]) was implemented in the heuristic search for the best ML tree applying the Tree Bisection and Reconnection (TBR) branch-swapping algorithm using PAUP*4.0 [107], starting with a neighbor-joining (NJ) tree constructed under the Hasegawa–Kishino–Yano (HKY85) model of evolution [108]. The robustness of the NJ phylogeny was assessed by bootstrap analysis with 1000 rounds of replication.

Genetic analyses were performed on gp160 sequences starting at nucleotide position 91, and for gp160 protein analyses starting at amino acid residue 31, thereby excluding the signal peptide. PNGS, and NXT and NXS motifs were identified using *N*-glycosite [109] at the Los Alamos HIV database website (http://www.hiv.lanl.gov/content/sequence/GLYCOSITE/glycosite.html). Overlapping PNGS (NN[TS][ST]) were included by *N*-glycosite as one PNGS, and NPS or NPT motifs were excluded.

### PBMC-based replication assays

To determine the viral replication rates of the different clonal viral variants, 2 × 10^6^ PHA-stimulated PBMCs from ten healthy uninfected donors were inoculated with 100 50 % tissue culture infective doses (TCID_50_) for 2 h at 37 °C in a shaking water bath, in a total volume of 2 ml. Subsequently, the PBMCs were washed with 10 ml of IMDM supplemented with 10 % fetal calf serum (FCS), and cultured in IMDM supplemented with 10 % FCS, 20 U/ml recombinant interleukin-2 (rIL-2), 5 μg/ml polybrene, 5 μg/ml ciproxine, 100 U/ml penicillin and 100 μg/ml streptomycin at a cell density of 1 × 10^6^ per ml in a humified CO_2_-incubator at 37 °C. Experiments were performed in duplicate. At 5, 8, 11 and 14 days after inoculation, 1 ml 1 × 10^6^ fresh PHA-stimulated PBMCs were added. Each day samples for p24 determination were taken and virus spread was analyzed with an in-house p24 antigen ELISA [110].

### PBMC-based neutralization assays

For measuring neutralization, virus (30 TCID_50_ in 25 μl when sera were tested, 20 TCID_50_ of virus in 50 μl when bNAbs were tested) was incubated for 1 h at 37 °C with threefold serial serum dilutions starting at a dilution of 1:50, or threefold serial bNAb dilutions. The starting concentration of bNAbs was 25 μg/ml for b12 and 2G12, 10 μg/ml for 8ANC195 and 5 μg/ml for VRC01, 12A21, PG9, PG16, PGT145, PGT121. PGT126, PGT128 and PGT135. bNAbs were obtained from Herman Katinger, Peter Kwong, Michel Nussenzweig and John Mascola, directly or through the AIDS reagent program. Subsequently, 1 × 10^5^ PHA-stimulated PBMCs were added. After an incubation of 4 h, PBMCs were washed once with 150 μl phosphate-buffered saline (PBS), put in 150 μl IMDM supplemented with 10 % FCS (inactivated at 56 °C for 30 min), 20 U/ml rIL-2, 5 μg/ml polybrene, 5 μg/ml ciproxine, 100 U/ml penicillin and 100 μg/ml streptomycin, and cultured in a humified CO_2_-incubator at 37 °C for 11 days. On days 7 and 11 virus production was measured by p24 ELISA [110]. The percent neutralization was calculated by determining the reduction in p24 production in the presence of serum or bNAbs compared to the cultures with virus only. 50 % inhibitory dose neutralization dilution (ID_50_ values; sera) and IC_50_ values (bNAbs) were determined by linear regression. For calculations, viruses with ID_50_ or IC_50_ values below the lowest Ab concentration or above the highest Ab concentration tested were assigned the lowest or highest Ab concentration tested. For viruses that were not inhibited by the 1:50 serum dilution, we assumed that 50 % inhibition would have occurred at a 1:25 serum dilution.

### Viral infection of TZM-bl cells

The TZM-bl reporter cell line stably expresses high levels of CD4-receptor and the HIV-1 coreceptors CCR5 and CXCR4 and contains the luciferase and β-galactosidase genes under control of the HIV-1 LTR promoter [111, 112]. The line was obtained through the National Institutes of Health AIDS Research and Reference Reagent Program from Dr. John C. Kappes, Dr. Xiaoyun Wu, and Tranzyme Inc., Durham, NC. TZM-bl cells were maintained in DMEM containing 10 % FCS, MEM nonessential amino acids, and penicillin/streptomycin (both at 100 U/ml). One day prior to infection, TZM-bl cells (1.7 × 10^4^ cells) were seeded in a 96-well plate in DMEM containing 10 % FCS, MEM nonessential amino acids, and penicillin/streptomycin (both at 100 U/ml) and incubated at 37 °C in an atmosphere containing 5 % CO_2_. A fixed amount of virus (500 pg) was added to the TZM-bl cells (70–80 % confluency) in the presence of 400 nM saquinavir (Roche Applied Science) and 40 μg/ml DEAE, in a total volume of 200 μl. Two days post-infection, the medium was removed, cells were washed once with PBS and subsequently lysed in Reporter Lysis buffer (Promega, Madison, WI). Luciferase activity was measured using the luciferase assay kit (Promega) and a Glomax luminometer according to the manufacturer’s instructions (Turner BioSystems, Sunnyvale, CA). All infections were performed in quadruplicate. Uninfected cells were used to correct for background luciferase activity.

### TZM-bl based neutralization assays

N332-glycan dependent neutralization was assessed by comparing neutralization of JRCSF, 92BR020 and MGRM-C-026 Env-pseudotyped viruses and their N332A glycan knock-out variants with D16916 serum taken 14 months post-SC, and using BG505 Env-pseudotyped virus and its T332N knock-in variant with serum taken 30 months post-SC. Substitutions were made using the Quikchange mutagenesis kit (Agilent, Santa Clara, CA) [111]. The experiments were carried out as described previously [37]. In summary, one day prior to infection, TZM-bl cells were plated on a 96-well plate in DMEM containing 10 % FCS, 1 × MEM nonessential amino acids, penicillin and streptomycin (both at 100 U/ml), and incubated at 37 °C in an atmosphere containing 5 % CO_2_ for 48 h. Virus (500 pg) was incubated for 30 min at room temperature with threefold serial dilutions of serum, starting at 1:37.5 or 1:40 (after the addition of virus). This mixture was added to the cells and 40 µg/ml DEAE, in a total volume of 200 µl. Two days later, the medium was removed. The cells were washed once with PBS (150 mM NaCl, 50 mM sodium phosphate, pH 7.0) and lysed in Reporter Lysis Buffer (Promega, Madison, WI). Luciferase activity was measured using a Luciferase Assay kit (Promega, Madison, WI) and a Glomax Luminometer according to the manufacturer’s instructions (Turner BioSystems, Sunnyvale, CA). Uninfected cells were used to correct for background luciferase activity. Nonlinear regression curves were determined and IC_50_ values were calculated using a sigmoid function in Graphpad Prism v5.01.

### Construction of HIV-1_LAI_ molecular clones and generation of viruses

A slightly modified version of the full-length molecular clone HIV-1_LAI_ (pLAI; [113]) was used as the basis for introducing mutations. These modifications included the S162T, G167D, V169K and E172V substitutions, designed to restore the epitopes for bNAbs PG9 and PG16 [114]. This modified pLAI was further changed to contain V1 elongations based on the sequences observed in individual D16916 as outlined in the results section and using previously described methods [115]. Briefly, mutant *env* genes were generated in pRS1 plasmid and cloned into pLAI as SalI-BamHI fragments. Mutations, deletions, and insertions were generated using the QuikChange mutagenesis kit (Stratagene, La Jolla, CA), and the integrity of all plasmids was verified by sequencing.

Virus stocks were generated by transfecting HEK293T cells with 4 μg full-length pLAI (or mutant pLAI), using the lipofectamine method [62]. HEK293T cells were purchased from the American Type Culture Collection and cultivated in DMEM containing 10 % FCS, 1 % streptomycin and 75 mM NaHCO_3_. Three days after transfection, virus-containing culture supernatants were harvested, filtered, and stored at −80 °C. Virus was quantitated by p24 ELISA [116] and viruses were normalized based on p24.

### SupT1-based replication and evolution assays

SupT1 cells were maintained in DMEM supplemented with 10 % FCS, 100 U/ml penicillin and 100 μg/ml streptomycin as described previously [117], and transfected by electroporation as described previously [118]. Evolution experiments were performed essentially as described before [115, 119]. A total of 5 × 10^6^ SupT1 cells were transfected with 5 or 20 μg pLAI (or mutant pLAI), and virus spread was monitored by visual inspection for the appearance of syncytia and by p24 ELISA as indicators of virus replication. Viruses were cultured for ∼3 months and decreasing amounts of supernatant were passaged cell-free onto uninfected cells when virus replication was apparent. At regular intervals, cells and filtered supernatant were stored at −80 °C for subsequent genotypic and phenotypic analysis and virus production was quantitated by p24 ELISA. When a putative faster-replicating virus was identified, DNA was extracted from infected cells using the QIAamp DNA mini kit (Qiagen, Valencia, CA), and the complete proviral *env* sequences were PCR amplified and sequenced.

### Statistical analyses

Statistical analyses were performed using Graphpad Prism v5.01. Differences between neutralizing responses were assessed using two-tailed Mann–Whitney tests, while differences in sequence (number of amino acids and PNGS) were analyzed by Student’s t-tests. Correlations between gp160 sequences with p24 production per virus isolate per day and with neutralizing responses were evaluated with a linear regression model. Differences and correlations were considered statistically significant when p values were ≤0.05.

## Additional files

10.1186/s12977-016-0279-4 Longitudinal sampling, CD4^+^ T-cell count and viral RNA load during the course of infection of individual D16916. Arrows indicate the time points from which clonal HIV-1 variants were isolated (black), and from which serum was obtained for autologous and heterologous neutralization experiments (light and dark grey, respectively). CD4^+^ T-cell count (red) and HIV-1 RNA viral load measurements (black).
10.1186/s12977-016-0279-4 Neutralization sensitivity of viral isolates for bNAbs. Clonal HIV-1 variants from 7 and 11 months post-SC were tested for their neutralization sensitivity for mAbs PGT121, PGT126, PGT128, 2G12, VRC01, PG9 and 8ANC195. The IC_50_ values are plotted and the horizontal bars represent the median IC_50_ value.
10.1186/s12977-016-0279-4 Maximum-likelihood tree of longitudinal gp160 *env* sequences from isolated clonal HIV-1 variants. Clonal HIV-1 env sequences from 7, 11, 19, 22 and 30 months post-SC were aligned and a Maximum-likelihood (ML) tree was constructed, and are indicated in green, blue, red, purple and orange, respectively. Analyses were done with total gp160 sequences, including gaps, because for this individual the V1, harboring the additional cysteines, was of great interest.
10.1186/s12977-016-0279-4 bNAb epitopes and the corresponding viral sequence alignment. Amino acid sequences of bNAb epitopes for isolates from 7 and 11 months post-SC. Contact residues (<5.0 Å distance from gp120 residues based on the crystal structures of gp120-bNAb co-complexes [60, 121, 122, 124] are indicated with black dots for (A) PGT135, (B) b12 (grey), 12A21 and (C) PG16.
10.1186/s12977-016-0279-4 Schematic representation of the V1V2 loop observed in D16916. Schematic of the V1V2 loops based on Bontjer et al. [125]. Cysteine residues and disulfide bridges are indicated in red. The variable loops are depicted in yellow and the conserved bridging sheet β-strands 2 and 3, are indicated in green. (A) V1V2 loop containing the normal number of cysteine residues. (B) V1V2 loop with 2 additional cysteine and adjacent residues, forming an “oven mitt” structure. (C) V1V2 loop with 2 additional cysteine and adjacent residues, resulting in a longer V1 compared to (A). (D-E) V1V2 with 4 additional cysteine and adjacent residues forming two “oven mitt” structures, (F) or an alternative structure.

## Abbreviations

HIV-1: human immunodeficiency virus type-1
bNAbs: broadly neutralizing antibodies
Env: envelope glycoprotein complex
ACS: Amsterdam Cohort Studies on HIV-1 infection and AIDS
NAbs: neutralizing antibodies
C: conserved regions
V: variable regions
PNGS: potential *N*-linked glycosylation site(s)
post-SC: post-seroconversion
WT: wild-type
CD4bs: CD4 binding site
CA: capsid
IDU: intravenous drug use
HLA: human leukocyte antigen
PHA: phytohemagglutinin
PBMCs: peripheral blood mononuclear cells
ML: maximum likelihood
hLRT: hierarchical likelihood ratio test
TBR: tree bisection and reconnection
NJ: neighbor-joining
HKY85: Hasegawa–Kishino–Yano
TCID_50_: 50 % tissue culture infective dose
FCS: fetal calf serum
ELISA: enzyme linked immunosorbent assay
PBS: phosphate-buffered saline
rIL-2: recombinant interleukin-2
IC_50_: 50 % inhibitory concentration
ID_50_ dilution: 50 % inhibitory dilution
