# Supplementary figures and images for: HIV-1 escapes from N332-directed antibody neutralization in an elite neutralizer by envelope glycoprotein elongation and introduction of unusual disulfide bonds
Source: Retrovirology. 2016 Jul 7;13:48. doi: 10.1186/s12977-016-0279-4 (PMC4936165; doi:10.1186/s12977-016-0279-4)

Additional file 1: Fig. S1

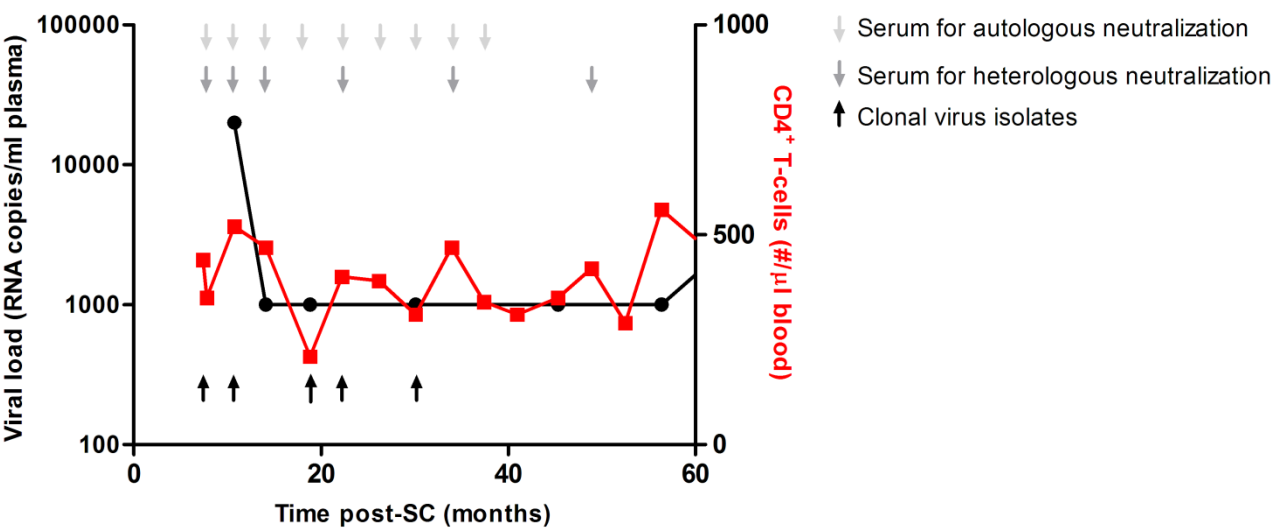

Supplement: Supplementary file 1 — 10.1186/s12977-016-0279-4 Longitudinal sampling, CD4+ T-cell count and viral RNA load during the course of infection of individual D16916. Arrows indicate the time points from which clonal HIV-1 variants were isolated (black), and from which serum was obtained for autologous and heterologous neutralization experiments (light and dark grey, respectively). CD4+ T-cell count (red) and HIV-1 RNA viral load measurements (black). [file 12977_2016_279_MOESM1_ESM.pdf]

Additional file 2: Fig. S2

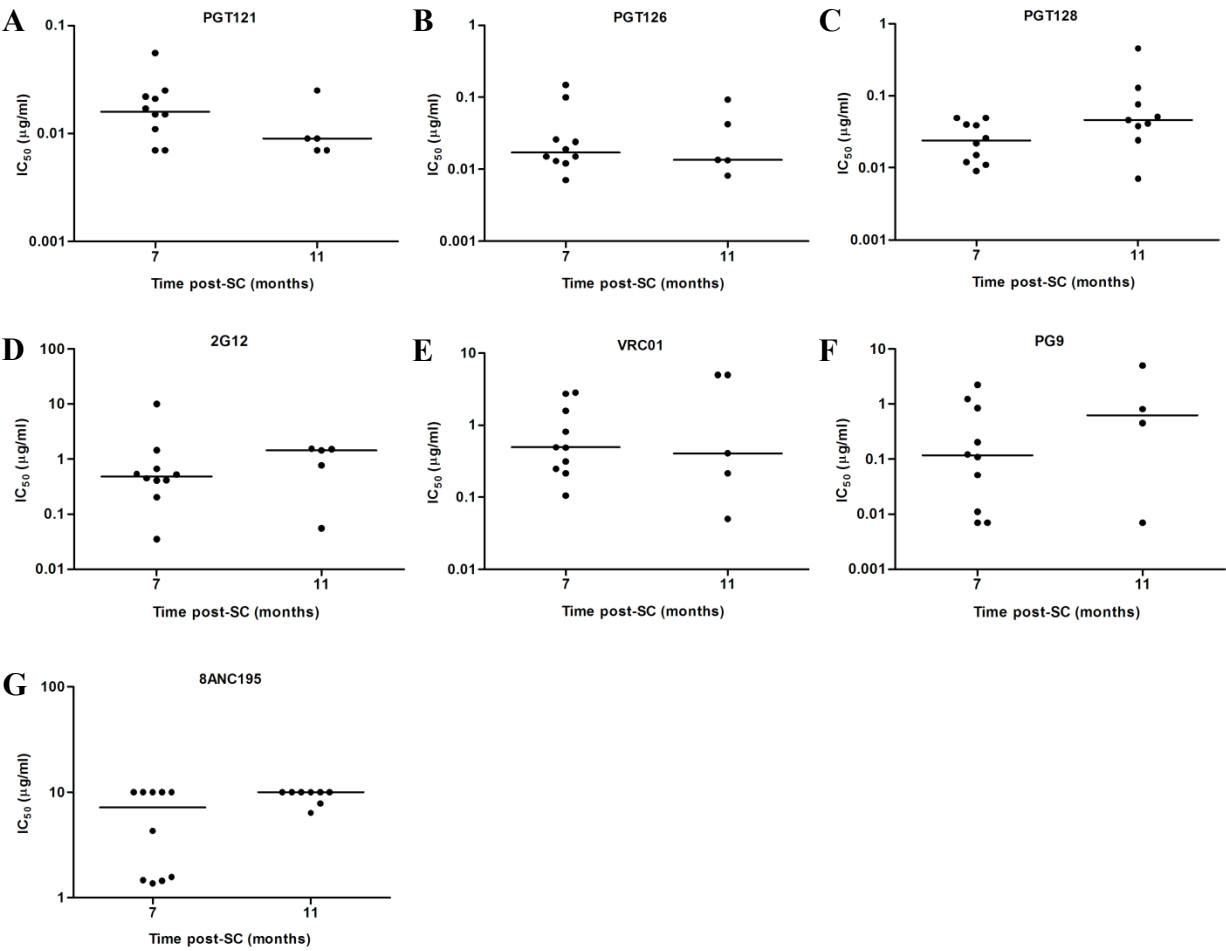

Supplement: Supplementary file 2 — 10.1186/s12977-016-0279-4 Neutralization sensitivity of viral isolates for bNAbs. Clonal HIV-1 variants from 7 and 11 months post-SC were tested for their neutralization sensitivity for mAbs PGT121, PGT126, PGT128, 2G12, VRC01, PG9 and 8ANC195. The IC50 values are plotted and the horizontal bars represent the median IC50 value. [file 12977_2016_279_MOESM2_ESM.pdf]

Additional file 3: Fig. S3

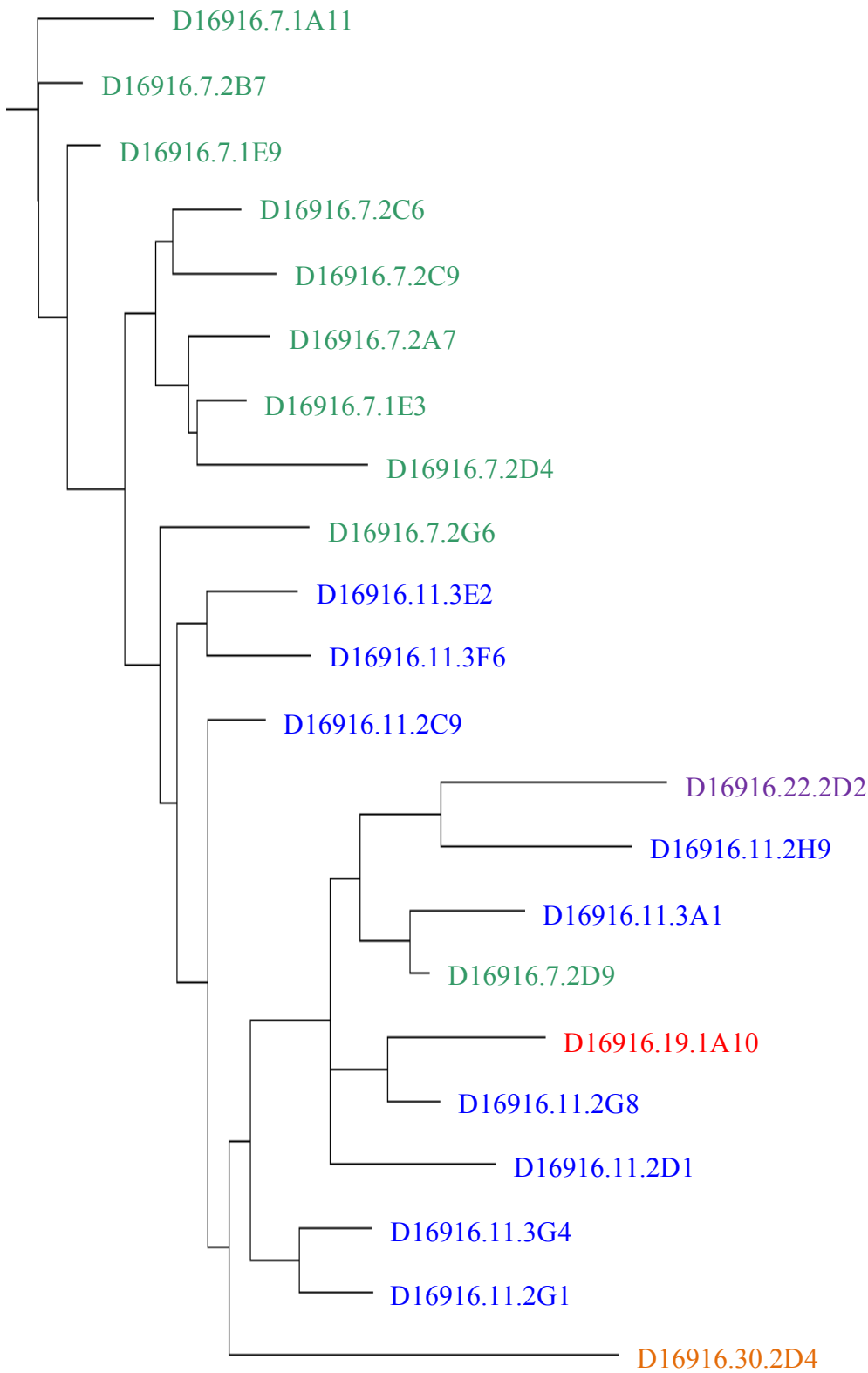

Supplement: Supplementary file 3 — 10.1186/s12977-016-0279-4 Maximum-likelihood tree of longitudinal gp160 env sequences from isolated clonal HIV-1 variants. Clonal HIV-1 env sequences from 7, 11, 19, 22 and 30 months post-SC were aligned and a Maximum-likelihood (ML) tree was constructed, and are indicated in green, blue, red, purple and orange, respectively. Analyses were done with total gp160 sequences, including gaps, because for this individual the V1, harboring the additional cysteines, was of great interest. [file 12977_2016_279_MOESM3_ESM.pdf]

# Additional file 4: Fig. S4

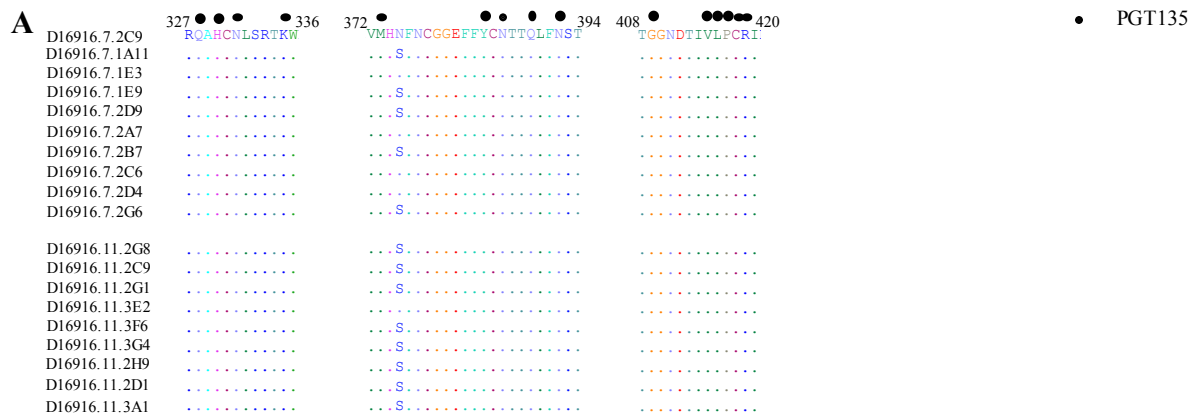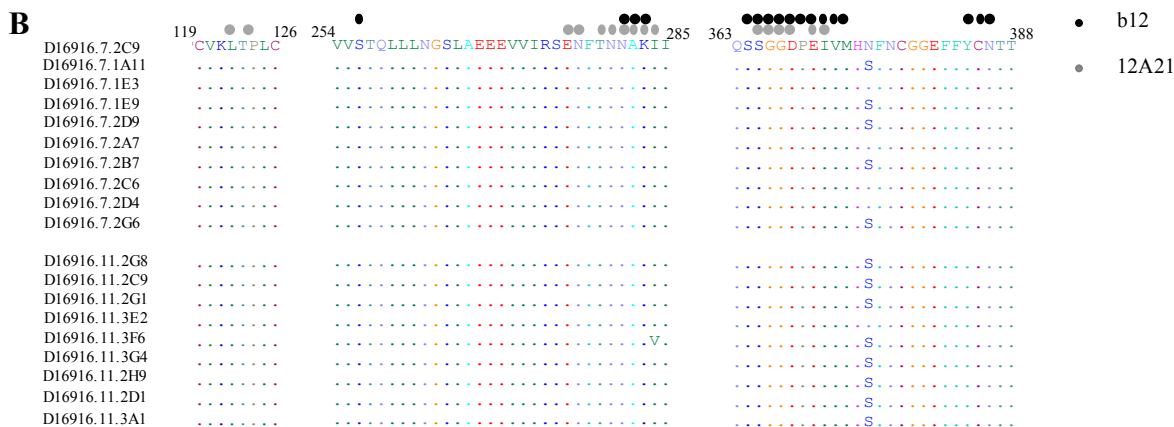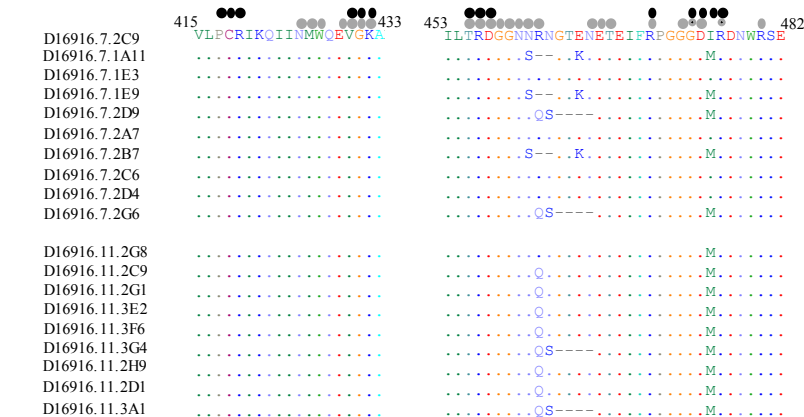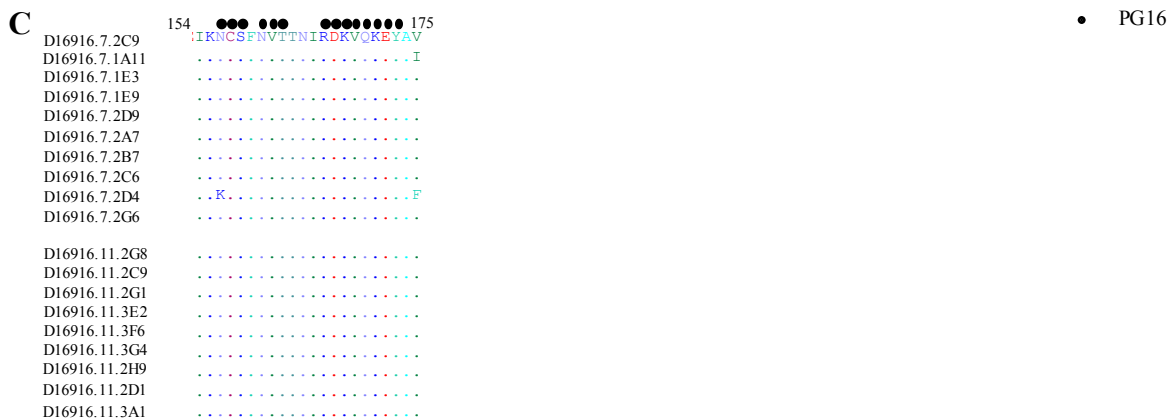

Supplement: Supplementary file 4 — 10.1186/s12977-016-0279-4 bNAb epitopes and the corresponding viral sequence alignment. Amino acid sequences of bNAb epitopes for isolates from 7 and 11 months post-SC. Contact residues (<5.0 Å distance from gp120 residues based on the crystal structures of gp120-bNAb co-complexes [60, 121, 122, 124] are indicated with black dots for (A) PGT135, (B) b12 (grey), 12A21 and (C) PG16. [file 12977_2016_279_MOESM4_ESM.pdf]

Additional file 5: Fig. S5

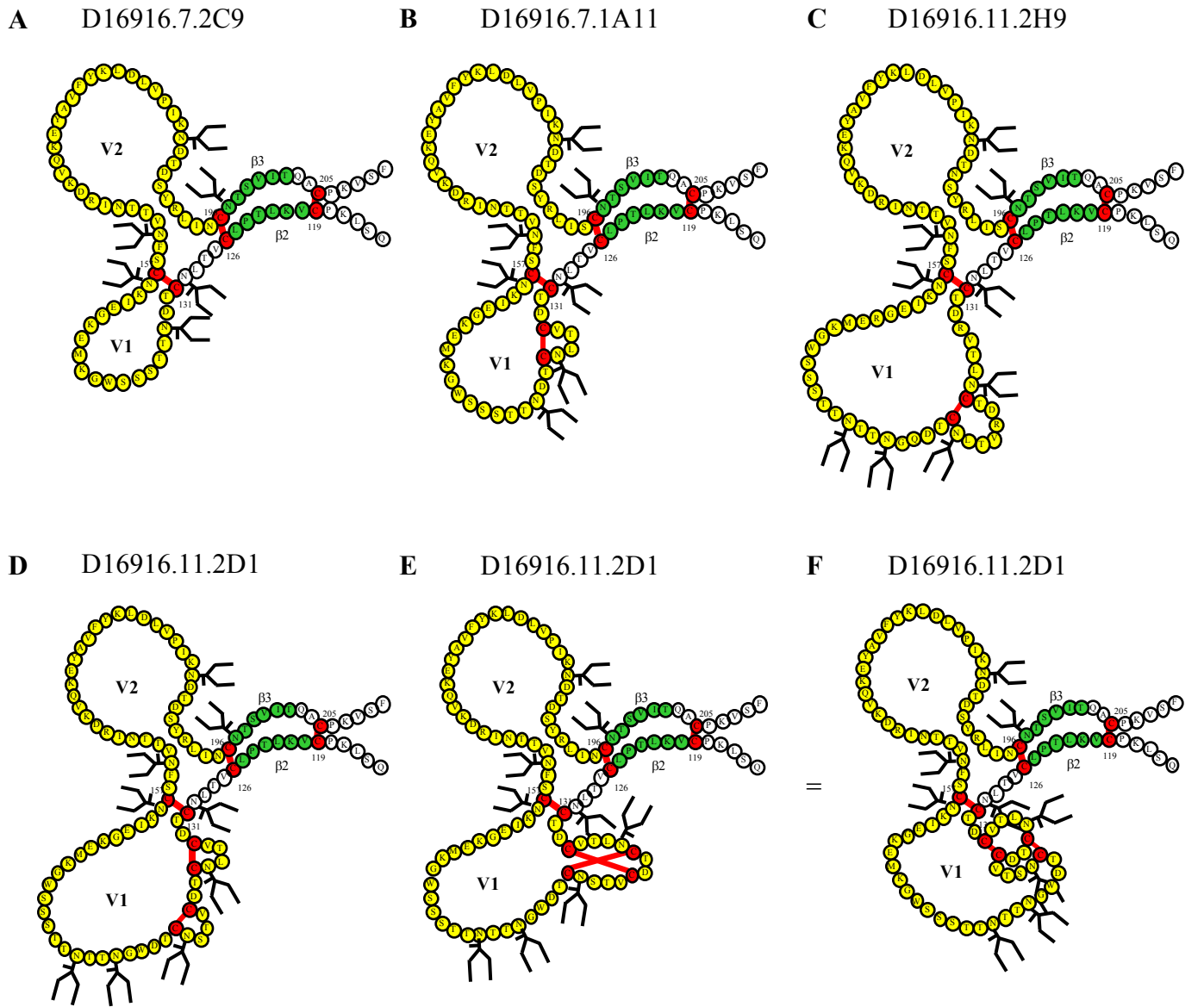

Supplement: Supplementary file 5 — 10.1186/s12977-016-0279-4 Schematic representation of the V1V2 loop observed in D16916. Schematic of the V1V2 loops based on Bontjer et al. [125]. Cysteine residues and disulfide bridges are indicated in red. The variable loops are depicted in yellow and the conserved bridging sheet β-strands 2 and 3, are indicated in green. (A) V1V2 loop containing the normal number of cysteine residues. (B) V1V2 loop with 2 additional cysteine and adjacent residues, forming an “oven mitt” structure. (C) V1V2 loop with 2 additional cysteine and adjacent residues, resulting in a longer V1 compared to (A). (D-E) V1V2 with 4 additional cysteine and adjacent residues forming two “oven mitt” structures, (F) or an alternative structure. [file 12977_2016_279_MOESM5_ESM.pdf]
